# Letter from the Editor in Chief

**DOI:** 10.19102/icrm.2021.120707

**Published:** 2021-07-15

**Authors:** Moussa Mansour


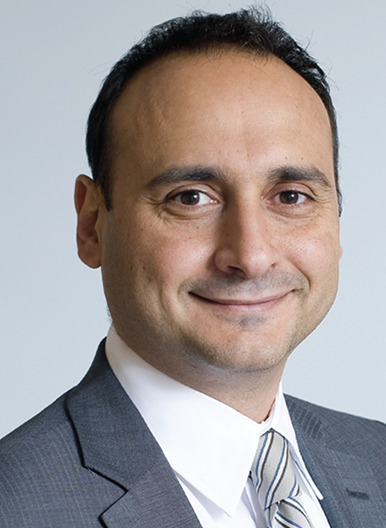


Dear readers,

In this issue of *The Journal of Innovations in Cardiac Rhythm Management*, Alqam et al.^1^ assess the discontinuation of anticoagulation after typical flutter ablation. Although retrospective and nonrandomized, their study offers important information that can help physicians managing patients with atrial flutter.

Meanwhile, no randomized studies have investigated the continuation versus cessation of oral anticoagulation after catheter ablation for atrial fibrillation (AF). A 2017 expert consensus statement on catheter and surgical ablation of AF^2^ recommended adherence to AF anticoagulation in patients with a history of AF ablation, regardless of procedural success or failure. Continuous or frequent electrocardiographic monitoring to screen for AF recurrence should also be considered in patients in whom anticoagulation discontinuation is being weighed based on patient values and preferences.^2^ Some well-designed nonrandomized studies^3–8^ have found that catheter ablation for AF reduces the risk of stroke; stopping anticoagulation after successful ablation can also be safely accomplished in some patients if careful rhythm monitoring is performed.

The field of cardiac rhythm monitoring has rapidly expanded in recent years. I believe the discontinuation of anticoagulation after AF ablation can be safely accomplished in selected patients when paired with careful rhythm monitoring; however, randomized clinical studies that consider different monitoring practices and groups of patients remain critical.

Sincerely,


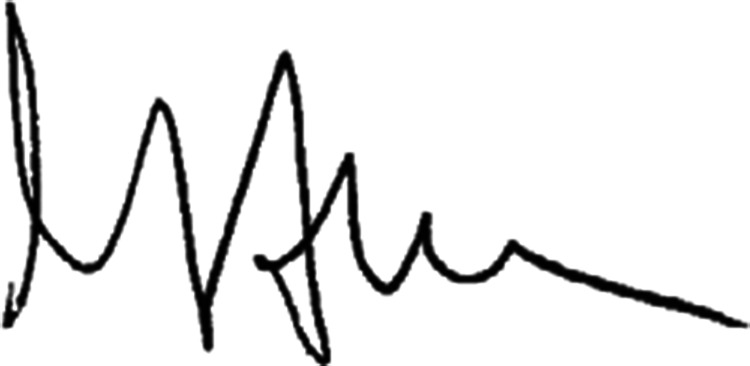


Moussa Mansour, md, fhrs, facc

Editor in Chief

*The Journal of Innovations in Cardiac Rhythm Management*

MMansour@InnovationsInCRM.com

Director, Atrial Fibrillation Program

Jeremy Ruskin and Dan Starks Endowed Chair in Cardiology

Massachusetts General Hospital

Boston, MA 02114
